# Population norms for the EQ-5D-3L: a cross-country analysis of population surveys for 20 countries

**DOI:** 10.1007/s10198-018-0955-5

**Published:** 2018-02-14

**Authors:** M. F. Janssen, A. Szende, J. Cabases, J. M. Ramos-Goñi, G. Vilagut, H. H. König

**Affiliations:** 1 Section Medical Psychology and Psychotherapy, Department of Psychiatry, Erasmus MC, PO Box 2040, 3000 CA Rotterdam, The Netherlands; 2 EuroQol Group, Rotterdam, The Netherlands; 3 Global Health Economics and Outcomes Research, Covance, Leeds, UK; 4 Department of Economics, Public University of Navarra, Pamplona, Spain; 5 IMIM (Institut Hospital del Mar d′Investigacions Mèdiques), Barcelona, Spain; 6 CIBER Epidemiología y Salud Pública (CIBERESP), Madrid, Spain; 7 Department of Health Economics and Health Services Research, University Medical Center Hamburg-Eppendorf, Hamburg, Germany

**Keywords:** Health state values, EQ-5D, Population norms, Health-related quality of life, I10, I30, J11, H51

## Abstract

This study provides EQ-5D population norms for 20 countries (*N* = 163,838), which can be used to compare profiles for patients with specific conditions with data for the average person in the general population in a similar age and/or gender group. Descriptive EQ-5D data are provided for the total population, by gender and by seven age groups. Provided index values are based on European VAS for all countries, based on TTO for 11 countries and based on VAS for 10 countries. Important differences exist in EQ-5D reported health status across countries after standardizing for population structure. Self-reported health according to all five dimensions and EQ VAS generally decreased with increasing age and was lower for females. Mean self-rated EQ VAS scores varied from 70.4 to 83.3 in the total population by country. The prior living standards (GDP per capita) in the countries studied are correlated most with the EQ VAS scores (0.58), while unemployment appeared to be significantly correlated in people over the age of 45 only. A country’s expenditure on health care correlated moderately with higher ratings on the EQ VAS (0.55). EQ-5D norms can be used as reference data to assess the burden of disease of patients with specific conditions. Such information, in turn, can inform policy-making and assist in setting priorities in health care.

## Introduction

EQ-5D is a standardized health-related quality of life questionnaire developed by the EuroQol Group in order to provide a simple, generic measure of health for clinical and economic appraisal [1]. Applicable to a wide range of health conditions, it provides a simple descriptive profile, a self-report visual analogue scale (EQ VAS) and an index value (‘utility’) for health status that can be used in the clinical and economic evaluation of health care as well as in population health surveys.

Since EQ-5D was first developed, a substantial amount of research has been carried out worldwide using the instrument [2]. Among this research were surveys conducted in various countries that measured the health-related quality of life of the general population [3]. These EQ-5D surveys have been informative in providing new data on population health characteristics, complementing the traditionally collected morbidity and mortality data.

Although recently an expanded five-level version of the EQ-5D instrument (EQ-5D-5L) has become available and was translated for use across countries, the general population survey datasets available in the EuroQol archive that were analyzed in this study were still based on the original three-level version of the EQ-5D (EQ-5D-3L), here referred to as EQ-5D.

The purpose of the current study is to present EQ-5D population norms for 20 countries, including reported problems by the five EQ-5D dimensions, self-reported EQ VAS ratings (by country, age, and gender), and EQ-5D index values (by country, age, and gender). The index values, presented in country-specific value sets, are a major feature of the EQ-5D instrument. EQ-5D value sets are typically obtained using representative samples of the general public, thereby ensuring that they represent the societal perspective, traditionally based on visual analogue scale (VAS) and time trade-off (TTO) valuation techniques. Apart from VAS- and TTO-based value sets, we also included the European VAS-based value set as a common metric for all countries. We hypothesized that reported health problems will increase by age and will be higher for females. Cross-country analyses of population health based on EQ-5D are presented with the aim of exploring which macroeconomic factors are associated with the self-reported health of the population. Additionally, we performed exploratory analyses on comparing the different value sets.

## Methods

### Data

Datasets per country were generally made available through the central data archive of the EuroQol Research Foundation. Countries included in the analysis were: Argentina, Belgium, China, Denmark, England, Finland, France, Germany, Greece, Hungary, Italy, Korea, the Netherlands, New Zealand, Slovenia, Spain, Sweden, Thailand, United Kingdom, and the United States [4–18]. For two countries (Argentina and China), the dataset transfer to the central archive was not possible. For these countries, data were analyzed locally by two collaborating researchers (FA, SS, respectively). All of the surveys included the standardized three-level version of EQ-5D, using the appropriate language version in each country. The Dutch, Swedish, and Finnish versions were translated in 1987 according to a ‘simultaneous’ process while the remaining versions were translated according to the EuroQol Group’s translation protocol based on international guidelines.

Table 1 provides a detailed account of the data by country. All datasets were collected in representative samples of the general population for each country. The datasets were structured in a standardized format to facilitate comparative research, although each survey also has its own characteristics and variables specific to the individual research context in which they were conducted. The datasets captured for the current analyses include observations on 163,838 individuals. Sampling weights were applied for Belgium, England, France, Germany, Italy, the Netherlands, and Spain according to a stratified, multistage, cluster-area, probability-sample design [5]. For the United States, sampling weights were applied resulting from a sampling design including stratification, clustering, multiple stages of selection, and oversampling of minority populations [18].

**Table 1:** National representative EQ-5D population surveys

Country	Source	Sample size	Data collection	Survey method
Argentina	Second National Survey of Risk Factors, 2005 [4]	41,392	2005	Face-to-face interviews on the representative 2005 Risk Factors Survey on a random selection of households
Belgium	ESEMED, König et al. [5]	2411	2001–2003	Computer-assisted home interviews on a nationally representative sample of the noninstitutionalized general adult population as part of the European Study of the Epidemiology of Mental Disorders (ESEMeD), using a stratified probability sample design
China	Sun et al. [6]	8031	2010	Face-to-face interviews on the representative 2010 Household Health Survey (HHS), using a stratified, multi-stage, clustered, random sampling design
Denmark	Sørensen et al. [7]	16,861	2000–2001	Face-to-face interviews on three representative national surveys based on randomized samples, including a national health interview survey undertaken by the National Institute of Public Health (SUSY-2000), a health survey undertaken in Funen County (Funen data set) and a national health survey undertaken by the University of Southern Denmark (SDU data set) with a total of 22,486 individuals
England	Health Survey for England 2008 [8]	14,763	2008	Computer-assisted interviews on a randomly selected sample of households in England
Finland	Saarni et al. [9]	8028	2000	Face-to-face interviews on the Health 2000 survey sample, which is a representative survey of the Finnish population aged 30 and over, following a two-stage, stratified, clustered sampling design
France	ESEMED, König et al. [5]	2892	2001–2003	Computer-assisted home interviews on a nationally representative sample of the noninstitutionalized general adult population as part of the European Study of the Epidemiology of Mental Disorders (ESEMeD), using a stratified probability sample design
Germany	ESEMED, König et al. [5]	3552	2001–2003	Computer-assisted home interviews on a nationally representative sample of the noninstitutionalized general adult population as part of the European Study of the Epidemiology of Mental Disorders (ESEMeD), using a stratified probability sample design
Greece	Yfantopoulous [10]	464	1998	Face-to-face interviews on a sample of 500 individuals selected from the general population, using quota sampling to ensure representativeness
Hungary	Szende and Nemeth [11]	5503	2000	Self-administered questionnaire during a personal interview on a random sample of 7000 people from the electoral registry
Italy	ESEMED, König et al. [5]	4709	2001–2003	Computer-assisted home interviews on a nationally representative sample of the noninstitutionalized general adult population as part of the European Study of the Epidemiology of Mental Disorders (ESEMeD), using a stratified probability sample design
Korea	Lee et al. [12]	1307	2007	Face-to-face interviews on a random sample of the South Korean residential registry
Netherlands	ESEMED, König et al. [5]	2367	2001–2003	Computer-assisted home interviews on a nationally representative sample of the noninstitutionalized general adult population as part of the European Study of the Epidemiology of Mental Disorders (ESEMeD), using a stratified probability sample design
New Zealand	Devlin et al. [13]	1327	1999	Postal survey on a randomized sample of 3000 New Zealanders selected from the electoral roll
Slovenia	Prevolnik Rupel and Rebolj [14]	742	2000	Postal survey on a randomized sample of 3000 people selected from the general population
Spain	ESEMED, König et al. [5]	5473	2001–2003	Computer-assisted home interviews on a nationally representative sample of the noninstitutionalized general adult population as part of the European Study of the Epidemiology of Mental Disorders (ESEMeD), using a stratified probability sample design
Sweden	Bjork et al. [15]	534	1994	Postal survey on a randomized sample of 1000 Swedish citizens selected from the general population from an address register
Thailand	Tongsiri et al. [16]	1409	2007	Face-to-face interviews on a random national sample provided by the national statistical office
United Kingdom	Kind et al. [17]	3395	1993	Face-to-face interviews on a random sample of 5324 individuals selected from the general population (based on the Postcode Address file) from England, Scotland, and Wales
United States	MEPS, Sullivan et al. [18]	38,678	2000–2002	Paper-and-pencil questionnaire among the Medical Expenditure Panel Survey participants, a nationally representative survey of the US civilian noninstitutionalized population. The research pooled 2000, 2001, and 2002 MEPS data on 23,839, 32,122, and 37,418 individuals, using a stratified, multistage, clustered sampling design

Surveys differed in methods of data collection and sample sizes. Some of the surveys were postal, while others were performed as part of a face-to-face interview or administered by telephone. The Argentinean dataset had the largest sample with over 41,000 respondents, while the Greek and the Swedish national surveys had the smallest sample of around 500 respondents.

### Methods of describing population norms

Population norm data were calculated for the five dimensions, self-rated EQ VAS, and EQ-5D index values for the total population, by gender, and the following age groups: 18–24, 25–34, 35–44, 45–54, 55–64, 65–74, and 75 + years. Aggregate EQ-5D dimension results were dichotomized, reporting the proportion of respondents scoring any problem on each dimension (the sum of the proportion of reported level-2 and level-3 problems). EQ-5D index value were calculated using the following value sets: European VAS value set for all countries, country-specific time trade-off (TTO) value set if available (11 countries), and country-specific VAS value set if available (10 countries).

The TTO method has played an important role in generating value sets for the EQ-5D as one of the most widely accepted preference elicitation methods in economic evaluation [19] and the method of choice in the first [20] and several subsequent large-scale EQ-5D valuation studies [21]. The VAS has become the other widely used valuation method to elicit preferences for the EQ-5D, including 9 countries. Note that the VAS valuation method needs to be distinguished from the EQ VAS, which is a self-reported rating of the respondents’ own health. The European VAS value set was constructed using data from 11 valuation studies in 6 countries: Finland (1), Germany (3), The Netherlands (1), Spain (3), Sweden (1), and the UK (2). This survey included sufficient data from different European regions to make the European VAS dataset moderately representative for Europe [22, 23]. Relevant information on the TTO- and VAS-based value sets, including the scoring algorithms, can be found in Szende et al. [21], Xie et al. [24], and Scalone et al. [25].

Results were tabulated in alphabetic order.

### Cross-country analysis

It is important to note that while results in each age group may be compared across countries, the total population scores cannot be compared directly, as they reflect the unique age structure within each country. Cross-country summary data for reported problems by the five dimensions and EQ VAS were estimated using a standardized population structure for all countries with national EQ-5D surveys. Standardization for age was performed to avoid bias due to the fact that some populations have a relatively higher proportion of elderly people. Age standardization of reported problems by dimension and EQ VAS were based on the European population structure using Eurostat data from 2010 [26], using the following proportions for each age group: 11% (18–24), 17% (25–34), 18% (35–44), 18% (45–54), 15% (55–64), 11% (65–74), and 10% (75 +).

To explore reasons for cross-country differences in EQ-5D data, correlations between country-specific EQ-5D data (five dimensions and self-rated EQ VAS) and country-specific macroeconomic indicators were calculated, including indicators of living standards and health system performance. Living standards were estimated by means of gross domestic product (GDP) per capita and unemployment rate. Indicators for health care system performance were health expenditure per capita and health expenditure as a percentage of GDP, number of hospital beds per 1000 people, and number of physicians per 1000 people. The indicators were selected on the basis of a presumed or possible relationship with self-reported health. Data were obtained from the World Health Organization Statistical Information System and the World Bank [27, 28]. The data were from 2010 or the closest year with available data (Table 2). An alternative set of macro data was also used to see how results might change when using macro data from the same year as the EQ-5D data collection, including variables on gross national income on purchasing power parity, unemployment rate, and health expenditure data.

**Table 2:** Country-specific macroeconomic indicators

	GDP per capita ($) 2010	Unemployment rate (%) 2010*	Health expenditure (% of GDP) 2010*	Health expenditure per capita ($) 2010*	Physicians per 1000 people 2004-2009
Argentina	9124	8.6	8.1	742	3.2
Belgium	43,006	8.3	10.7	4618	3.0
China	4433	4.3	5.1	221	1.4
Denmark	56,486	7.4	11.4	6422	3.4
France	39,170	9.3	11.9	4691	3.5
Germany	40,164	7.1	11.6	4668	3.5
Greece	25,832	12.5	10.2	2729	6.0
Hungary	12,863	11.2	7.3	942	3.1
Italy	33,787	8.4	9.5	3248	4.2
Korea	20,540	3.7	6.9	1439	2.0
Netherlands	46,623	4.5	11.9	5593	3.9
New Zealand	32,407	6.5	10.1	3279	2.4
Slovenia	22,898	7.2	9.4	2154	2.5
Spain	29,956	20.1	9.5	2883	3.7
Sweden	49,360	8.4	9.6	4710	3.8
Thailand	4614	1.2	3.9	179	0.3
United Kingdom	36,256	7.8	9.6	3503	2.7
United States	46,612	9.6	17.9	8362	2.4

*Data availability for last year varies in some countries

A non-parametric measure (Spearman rank correlation) was used to assess the association between self-reported health using EQ-5D and the above-mentioned indicators of living standards and health system performance. We expected that poorer populations will show more reported health problems than richer populations, and countries with a shorter life expectancy will also display more reported health problems. Generally, the positive association of good health with higher health expenditures probably rests on a common explanatory factor, i.e., wealth on the country level. As additional exploratory analysis, we performed linear regression analyses on macroeconomic indicators and mean VAS rating.

The inclusion of both the European VAS value set as well as country-specific VAS value sets allowed for exploring the impact of the preferences of a specific country, using the European VAS value set as a reference. The inclusion of the country-specific TTO value sets also allowed for exploring the effect of valuation method (VAS versus TTO). All data analyses were performed using SPSS version 19 and Stata version 12 statistical software packages.

## Results

### EQ-5D population norms

Results for reported problems along the five dimensions by gender for each country are presented in Table 3. As hypothesized, reported health problems were generally higher for females, with the exception of Slovenia. Problems with pain/discomfort were generally the most prevalent in each country, while problems with self-care were the least prevalent across countries. Thailand and Slovenia appeared to have generally high reported problems in all dimensions compared to other countries, while China and Korea showed the lowest reported problems. The pattern of reported problems across the five dimensions was rather similar across countries, although the absolute number of reported problems varies.

**Table 3:** Reported problems by five dimensions (proportions (%) of respondents scoring any problem, not standardized)

	Mobility		Self-care		Usual activities		Pain/discomfort		Anxiety/depression	
	Females	Males	Females	Males	Females	Males	Females	Males	Females	Males
Argentina	13	9	3	2	10	6	36	25	26	19
Belgium	15	10	5	3	15	10	31	26	8	5
China	6	4	3	3	6	4	13	8	10	7
Denmark	12	10	3	2	20	15	40	33	19	12
Finland	29	24	12	9	24	18	52	43	15	12
France	16	11	4	4	11	9	38	33	16	13
Germany	17	15	3	2	11	9	30	25	5	4
Greece	14	13	9	3	12	9	20	14	12	10
Hungary	23	16	7	6	17	12	45	32	42	27
Italy	12	9	5	2	12	7	31	22	11	6
Korea	9	3	1	0	6	2	27	16	23	12
Netherlands	13	9	4	2	16	10	38	30	4	2
New Zealand	20	20	4	5	22	21	41	40	24	18
Slovenia	28	32	14	14	33	33	48	47	38	34
Spain	16	11	6	3	14	8	27	17	10	5
Sweden	10	7	2	1	8	8	42	40	31	21
Thailand	28	24	8	9	22	23	68	62	51	43
UK	19	18	4	4	16	17	34	32	23	18
UK—England	21	18	6	5	18	15	37	33	22	16
United States	22	17	5	5	23	17	49	41	32	23

**Fig. 1:**
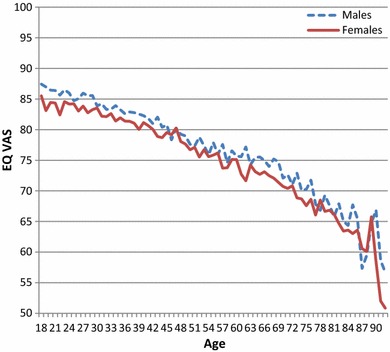
Self-rated mean EQ VAS by age and gender (pooled dataset*). * Including data for all countries except Argentina and China, which were not added to the central data archive

Table 4 shows results for self-rated EQ VAS scores for each country by age and gender and for the total population. EQ VAS ratings decreased with increasing age and were generally lower for females in all countries, which confirmed our hypotheses. Country-specific differences can be observed in the overall level of health (mean EQ VAS ratings), and to a lesser extent in the level of health decrease (age-slope). Korea displayed a very small age slope. The age slope was considerably higher in Southeastern Europe compared to Northwestern Europe. Gender differences were generally more pronounced with increasing age, and stronger for some countries while almost absent in others (New Zealand, Slovenia, and Thailand). For illustrative purposes, Fig. 1 shows the detailed age and gender pattern for the pooled dataset.

**Table 4:** Self-reported EQ VAS ratings by age group and total population (mean values, not standardized)

	18–24		25–34		35–44		45–54		55–64		65–74		75 +		Total	
	Females	Males	Females	Males	Females	Males	Females	Males	Females	Males	Females	Males	Females	Males	Females	Males
Argentina	80	84	78	81	76	79	73	76	69	70	66	70	61	64	74	77
Belgium	84	84	83	82	79	82	75	79	72	76	72	71	70	69	77	79
China	89	89	85	86	82	83	78	81	75	78	71	73	67	72	80	81
Denmark	86	86	88	88	86	86	83	82	81	82	77	80	77	76	84	84
France	84	83	84	82	78	79	78	78	75	73	67	70	60	64	76	77
Germany	86	85	84	84	83	82	79	78	73	73	66	72	60	61	76	78
Greece	82	85	87	85	83	86	77	79	59	77	70	65	47	61	78	80
Hungary	83	84	80	82	75	76	68	70	62	66	57	63	53	55	69	73
Italy	86	89	83	84	81	82	76	78	73	76	65	71	60	61	75	79
Korea	79	79	79	82	81	81	81	80	74	81	74	79	–	–	79	80
Netherlands	82	89	84	85	83	85	80	82	81	80	79	77	70	79	81	83
New Zealand	83	81	82	82	84	81	82	82	82	81	80	79	68	74	81	80
Slovenia	86	84	83	82	82	79	76	75	69	67	64	67	55	56	77	76
Spain	81	83	78	82	77	77	74	73	71	73	66	77	59	67	73	77
Sweden	83	86	87	86	85	88	84	83	78	80	78	84	66	84	82	84
Thailand	82	85	82	80	80	81	79	78	81	77	77	75	83	65	80	79
UK	86	87	87	87	86	87	82	82	80	84	77	78	74	73	82	83
United States	84	88	83	85	81	83	79	80	76	78	75	75	68	69	79	81

EQ-5D index norm values based on the European value set generally decreased with age, with values ranging from 0.814–0.990 in the youngest group, to 0.621–0.840 in the 75 + group. Corresponding EQ-5D index values in countries where TTO-based value sets were available ranged from 0.924 to 0.984 in the youngest group to 0.703–0.839 in the 75 + group. Finally, EQ-5D index values in countries where VAS-based value sets were available ranged from 0.869 to 0.962 in the youngest group to 0.498–0.817 in the 75 + group. Population norms based on the European VAS value set were generally higher than or similar to country-specific VAS value sets (except for Germany), while population norms based on country-specific TTO value sets tended to be higher compared to the same countries using country-specific VAS-based value sets (see Tables 5, 6).

**Table 5:** EQ-5D index value population norms by age group and total population (European VAS value set)

	18–24	25–34	35–44	45–54	55–64	65–74	75 +	Total
Argentina	0.907	0.889	0.869	0.849	0.829	0.796	0.724	0.856
Belgium	0.953	0.921	0.920	0.889	0.881	0.848	0.761	0.891
China	0.990	0.980	0.970	0.960	0.930	0.900	0.840	0.951
Denmark	0.914	0.914	0.881	0.861	0.845	0.818	0.753	0.866
Finland	N/A	0.919	0.891	0.853	0.805	0.762	0.573	0.815
France	0.924	0.921	0.883	0.893	0.836	0.804	0.756	0.872
Germany	0.950	0.949	0.943	0.908	0.881	0.838	0.771	0.902
Greece	0.979	0.972	0.957	0.916	0.817	0.793	0.739	0.913
Hungary	0.934	0.911	0.873	0.802	0.755	0.716	0.639	0.823
Italy	0.969	0.956	0.943	0.910	0.877	0.823	0.724	0.899
Korea	0.957	0.958	0.949	0.915	0.828	0.787	N/A	0.915
Netherlands	0.938	0.910	0.922	0.874	0.869	0.863	0.798	0.892
New Zealand	0.913	0.906	0.893	0.858	0.817	0.800	0.712	0.848
Slovenia	0.879	0.859	0.831	0.772	0.697	0.663	0.621	0.788
Spain	0.968	0.963	0.939	0.911	0.884	0.870	0.773	0.915
Sweden	0.888	0.893	0.868	0.835	0.813	0.836	0.701	0.851
Thailand	0.814	0.785	0.771	0.717	0.694	0.670	0.657	0.742
UK	0.934	0.922	0.905	0.849	0.804	0.785	0.734	0.856
UK—England	0.922	0.915	0.891	0.857	0.819	0.785	0.720	0.857
US	0.899	0.883	0.853	0.809	0.776	0.756	0.677	0.825

N/A: not available

**Table 6:** EQ-5D index value population norms by age group and total population (country-specific TTO and VAS value sets)

	18–24	25–34	35–44	45–54	55–64	65–74	75 +	Total
TTO value sets								
Argentina	0.951	0.936	0.919	0.898	0.874	0.835	0.756	0.902
Denmark	0.928	0.927	0.901	0.882	0.870	0.847	0.794	0.887
France	0.948	0.946	0.913	0.922	0.853	0.810	0.735	0.892
Germany	0.972	0.973	0.966	0.945	0.922	0.891	0.839	0.938
Italy	0.984	0.978	0.973	0.955	0.936	0.904	0.839	0.947
Korea	0.981	0.982	0.976	0.960	0.909	0.888	N/A	0.958
Netherlands	0.950	0.927	0.935	0.890	0.890	0.886	0.830	0.910
Spain	0.982	0.975	0.949	0.923	0.901	0.891	0.781	0.929
UK	0.940	0.927	0.911	0.847	0.799	0.779	0.726	0.856
UK—England	0.929	0.919	0.893	0.855	0.810	0.773	0.703	0.855
US	0.924	0.912	0.889	0.855	0.830	0.817	0.755	0.867
VAS value sets								
Argentina	0.928	0.911	0.888	0.867	0.837	0.793	0.712	0.871
Belgium	0.948	0.915	0.912	0.881	0.871	0.836	0.748	0.883
Denmark	0.885	0.884	0.845	0.822	0.799	0.766	0.691	0.826
Finland	N/A	0.909	0.878	0.835	0.781	0.738	0.583	0.800
Germany	0.962	0.966	0.962	0.937	0.915	0.882	0.817	0.930
New Zealand	0.890	0.883	0.869	0.827	0.782	0.763	0.672	0.818
Slovenia	0.869	0.841	0.794	0.712	0.619	0.554	0.498	0.738
Spain	0.969	0.963	0.939	0.912	0.883	0.866	0.761	0.914
UK	0.931	0.920	0.902	0.846	0.799	0.778	0.726	0.852
UK—England	0.922	0.914	0.888	0.854	0.814	0.775	0.706	0.853

N/A: not available

### Cross-country comparison

Table 7 shows the impact of age standardization of population norms, which were usually within a few percentage points of difference. Mean EQ VAS score varied from 70.4 to 83.3 in the total population. The largest differences between any two countries in reporting problems were 28.6, 12.7, 31.9, 53.7, and 43.8% in absolute terms along the five dimensions, respectively. Hungary reported the lowest EQ VAS ratings (70.4), followed by Korea (71.3), while Denmark (83.3) and the United Kingdom (82.8) reported the highest EQ VAS ratings. The highest proportion of problems on the five EQ-5D dimensions was reported by Slovenia and Thailand. It needs to be noted that while Hungary and Korea reported a lower mean EQ VAS than Slovenia and Thailand, generally more problems were reported in Slovenia and Thailand across the five dimensions. At the other end of the spectrum, China reported the lowest proportion of problems but reported average EQ VAS ratings, while Denmark and the UK reported the highest EQ VAS ratings and average proportions of problems. These results indicate that countries also differed in the overall level of health resulting from the more general EQ VAS question relative to the more specific questions on the EQ-5D dimensions.

**Table 7:** Self-reported EQ-5D results after age standardization (mean EQ VAS and proportions (%) of respondents scoring any problem)

	EQ-VAS	Mobility	Self-care	Usual activity	Pain/discomfort	Anxiety/depression
Argentina	73.9	13.3	3.7	9.8	33.9	23.8
Belgium	77.4	13.9	4.8	12.9	29.4	6.1
China	79.9	6.1	3.4	6.1	11.5	9.2
Denmark	83.3	11.5	2.8	18.6	37.0	16.2
France	76.3	14.4	4.6	10.7	35.8	14.5
Germany	77.2	17.2	3.1	10.5	27.8	4.5
Greece	76.5	17.2	8.3	13.7	20.4	11.2
Hungary	70.4	20.9	7.2	15.8	40.4	36.2
Italy	76.9	12.3	4.4	11.1	27.7	9.2
Korea	71.3	6.5	1.0	4.6	29.6	22.9
Netherlands	81.4	11.8	3.5	12.5	32.6	3.2
New Zealand	80.8	19.2	4.3	20.8	39.3	21.2
Slovenia	74.5	34.7	16.7	36.5	51.0	38.0
Spain	74.3	12.7	4.0	11.0	21.3	7.3
Sweden	82.5	11.3	2.5	9.6	42.5	26.4
Thailand	78.9	29.8	9.2	25.9	65.2	47.0
United Kingdom	82.8	18.2	4.3	16.2	33.1	20.9
US	79.3	19.3	3.7	18.3	48.0	22.4

Table 8 shows the association on the country level of the macroeconomic indicators and the EQ VAS rating and reported health problems. As hypothesized, the prior living standards (GDP per capita) and health expenditure per capita in the countries studied were correlated with the mean EQ VAS scores (0.58 and 0.55, respectively). Unemployment significantly correlated in people over the age of 45 only. The number of physicians did not correlate with better EQ VAS data (0.03). Contrary to our expectations, life expectancy did not result in any significant association.

**Table 8:** Spearman rank correlations between macroeconomic indicators and self-reported health (mean self-rated EQ VAS and proportion of any reported problem)

EQ-VAS						
Age group	GDP per capita	Unemployment	Health expenditure (% of GDP)	Health expenditure per capita	Physicians per 1000 people	Life expectancy
18–24	0.38	− 0.12	0.29	0.40	0.09	− 0.15
25–34	0.55*	− 0.06	0.44	0.53*	0.32	0.02
35–44	0.50*	− 0.26	0.35	0.47	0.18	0.09
45–54	0.49*	− 0.50*	0.29	0.48*	− 0.13	0.13
55–64	0.45	− 0.50*	0.26	0.45	− 0.25	0.13
65–74	0.47	− 0.48*	0.20	0.44	− 0.21	0.21
75 +	0.42	− 0.51*	0.17	0.37	− 0.24	0.02
Total	0.58*	− 0.35	0.39	0.55*	− 0.03	0.00

EQ-5D dimension						
	GDP per capita	Unemployment rate	Health expenditure (% of GDP)	Health expenditure per capita	Physicians per 1000 people	Life expectancy
Mobility	− 0.19	0.14	0.04	− 0.13	− 0.27	− 0.34
Self-care	− 0.35	0.26	− 0.14	− 0.35	− 0.05	− 0.19
Usual activities	0.08	− 0.03	0.13	0.09	− 0.24	− 0.27
Pain/discomfort	0.10	− 0.11	− 0.01	0.12	− 0.38	− 0.31
Anxiety/depression	− 0.38	− 0.04	− 0.51*	− 0.38	− 0.46	− 0.41

*p < 0.05

**Fig. 2:**
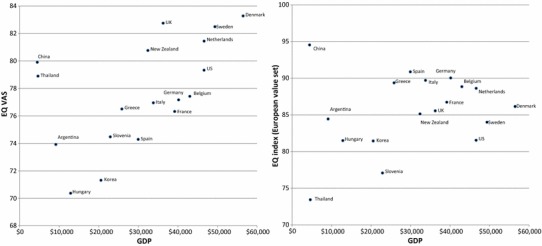
Self-rated EQ VAS and index values (European value set) according to GDP* per capita in 18 countries (mean values after age standardization). *GDP is the total value of all goods and services produced by an economy in 1 year

The positive relationship between living standards and self-reported EQ VAS was further examined and is graphically presented in Fig. 2. As shown, EQ VAS correlated well with a country’s GDP, although China and Thailand were outliers with an exceptionally low GDP (combined with relatively high EQ VAS scores). The European value set showed a more moderate correlation with GDP with only China as outlier and a smaller slope.

Linear regression analyses showed that GDP level explained 29% of EQ VAS at the country level (*p* = 0.02), but explained 67% of the EQ VAS when excluding ‘outliers’ China and Thailand. Health expenditure per capita was the only other statistically significant explanatory factor that explained 26% of the country mean VAS (*p* = 0.03). Another set of regression analyses, which used macro data from the year of EQ-5D data collection in each country on gross national income expressed in purchasing power parity in 2010 values, did not yield statistically significant results. However, health care expenditure remained a statistically significant factor (*p* = 0.03), explaining 27% of variation in the country mean VAS scores.

## Discussion

The current study generated population norms for self-rated EQ VAS and EQ-5D index values, and for self-reported problems on each of the five dimensions of the EQ-5D descriptive system for 20 countries, all classified by age. These EQ-5D norms are highly relevant for future research initiatives, as they can be used to compare EQ-5D data from patients to the average person in the general population of a certain country in a similar age (or gender) group, which also helps to identity the burden of the disease of patients or patient groups. This multi-country analysis is unique in terms of reporting EQ-5D data based on a standard methodology and allowing for comparisons across countries and explaining differences using macroeconomic indicators.

Our hypothesis on age and gender was confirmed by results for both the EQ VAS and reported problems on the five dimensions (where the age effect was visible through the index values, providing a summary score for the five dimensions). Cross-country differences occurred in EQ-5D outcomes in terms of the overall level of health but also in terms of the age slope, which was considerably higher in Southeastern Europe compared to Northwestern Europe. The overall patterns in each country regarding reported problems were spectacularly similar in terms of pain/discomfort being the most prevalent and self-care being the least prevalent problem. However, the actual rates of reporting problems differed widely across countries after accounting for demographic differences, and no consistent trend was observed on how countries score in terms of EQ VAS relative to morbidity reported along the five dimensions, which seems to indicate that the EQ VAS is measuring a different (or at least wider) health concept than the five dimensions of EQ-5D, or that countries differ in responses to the various dimensions. An obvious implication of these findings for multi-country studies with the EQ-5D is the need to factor in the country of origin of patients when analyzing and interpreting results.

In addition, when examining population norms for EQ-5D index values, results highlighted the importance of also taking into account the value set used to calculate the EQ-5D index when interpreting results or making comparisons across studies. Country-specific value sets are generally recommended for use in the corresponding country, while for comparative purposes, the European value set seems to be the most optimal choice. Country-specific value sets showed differences between valuation methods, which is consistent with previous evidence indicating that TTO methodology leads to higher values than VAS-based techniques [29].

The fact itself that self-reported health differs across countries is not unexpected. Previous studies, such as those based on categorical assessment of self-assessed health [30], or those based on generic quality of life questionnaires [31], found results that self-reported health differed across countries. These cross-country differences in the general level of health (EQ VAS) were at least partially explained by looking at macro data on the living standards and health system characteristics of each country. The analysis highlighted that it is the prior living standards of a country that mostly explain cross-country differences in self-reported health. Indeed, the result that GDP level explained 67% of EQ VAS at the country level when excluding two ‘outlier’ countries underlined the high importance of viewing self-reported health within a broader macroeconomic context. At the same time, health expenditure per capita was also quantified to be an important factor, one that policy-makers at a national level have more control over than determining annual GDP. In addition, while GDP showed a stronger correlation with VAS than health expenditure, a dollar unit of health expenditure had eight times the impact of a dollar unit of GDP on the country mean VAS scores (with coefficients of 0.0001 for GDP and 0.0008 for health expenditure). However, expenditure might be confused with GDP, since a high GDP might lead to higher health care expenditures, which in turn might influence the number and quality of interventions per capita, and consequently lead to better health in a population.

The most important limitation of this analysis relates to differences in samples across countries. While all samples were representative samples of the general population of each country, differences exist across study methodologies, such as sample size, administration method, purpose of data collection, and time of the data collection. While adjustments were made for sample structure, some of these factors may have influenced the comparability of the results. In particular, some surveys in the dataset archive were older, and limited evidence suggests that population norms may or may not change over time, depending on the country [3]. Non-response may have introduced a potential bias towards underestimation of self-reported health problems. Some countries applied a sampling design, whereas other countries did not, which might lead to a more accurate reflection of representativeness for the former. Although mode of administration might contribute to observed differences, a recent study showed equivalence between various modes of administration using the EQ-5D [32]. Further variability between countries might be caused by translations of the different versions of the EQ-5D. Another limitation is the use of the European population structure for age standardization, which might not be fully justified for the non-European countries, especially for China, where the population structure is quite different. Finally, influences due to reporting behavior heterogeneity, such as education, might also impact variability between self-reported health problems [33].

While results from these analyses can be used to compare profiles for patients with specific conditions or to assess the burden of disease in question, understanding inequalities in self-assessed health among the population is also important, but fell beyond the aims of this paper. However, more in-depth analyses on contributors to levels of population health could be important.

Finally, this manuscript focused on existing data from the three-level version of the EQ-5D instrument; however, a more refined version of the EQ-5D (EQ-5D-5L), which extends the three response levels in each dimension to five levels, has been introduced [34]. The extra levels are expected to lead to a much more accurate reflection of population health, especially in relation to mild health problems. Further important research in the field would be the reporting of population norms using the EQ-5D-5L version of the questionnaire.
